# An Obscure Case

**Published:** 1895-07

**Authors:** A. W. Clement


### An Obscure Case.

BY A. W. CLEMENT, V.S.

A BAY gelding, nine years old, one of a pair of coach horses, was taken suddenly ill in the afternoon. This animal had had only walking exercise at the halter for two weeks previous, as his mate had been ill with pneumonia.

On the morning of the day that he was taken sick the horse was particularly playful. At about four o'clock in the afternoon the coachman went to the livery-stable where they were kept, and noticed that the horse was very dull and breathed heavily. I was immediately sent for, and arrived at the stable about six o'clock. I found the animal greatly depressed, respiration labored, the heart beating tumultuously, and the horse hiccoughing incessantly. The pulse 60, and very irregular; temperature, 105°; lungs normal.

I suspected a severe attack of influenza, and had the animal removed to my infirmary. Broken doses of aconite soon had the heart under control, and the animal when left for the night seemed fairly comfortable. The next morning it was seen that his hind legs were quite stiff; respiration, 35; pulse, 60, weak, but regular; temperature, 105.2°. Stimulants and febrifuge prescribed and no attention given to stiffness of the hind legs, as it was concluded to be muscle-soreness, so often observed in cases of influenza, and which generally disappear as the disease progresses.

On the third day after admission to the hospital the fever had quite disappeared, but the respiration was still about 30, and the pulse 55 and fuller. The lameness had disappeared to a very great extent from the hind legs during the night, but extreme lameness with intense pain had developed in the near fore-leg. The flexor muscles were very tense, and he would not put his heel to the ground. When made to move he would hobble on three legs. The foot was thoroughly examined for a foreign body, but nothing of the kind was to be found. A diagnosis of acute rheumatism was made, and the animal was placed on salycilate of soda for twelve hours in doses of ʒj every three hours.

On the following day there was considerable swelling, hard and very tender on pressure, extending from the distal end of the humerus, half way up the scapula, and including the shoulder joint. This swelling lasted for about two weeks, then gradually disappeared; but when the horse left my place, some six weeks after he was taken sick, he was still unable to put his foot to the ground, and dragged that leg as if paralyzed. There was great atrophy of the shoulder-muscles, but at no time after the swelling had disappeared so that an examination could be made was there any evidence of fracture or dislocation. At no time during the period that he was under my care did he respond in the least to salycilates. Liniments and blisters were applied locally without any good results. I understand from the coachman that the horse has improved but slightly since he has been at pasture. The urine was examined some two or three times and found to be normal. There was no evidence of osteoporosis anywhere that could be detected. The flexors of the forearm were contracted most of the time, but occasionally they would relax when he would extend his foot as in navicular trouble. At no time was there any heat or tenderness about the foot, though poultices were applied often and the hoof kept in good condition. Was this lameness a purely local affair or was it of a rheumatic nature?

